# Current Evidence of 2019 Novel Coronavirus Disease (COVID-19) Ocular Transmission: A Systematic Review and Meta-Analysis

**DOI:** 10.1155/2020/7605453

**Published:** 2020-10-24

**Authors:** Kai Cao, Brad Kline, Ying Han, Gui-shuang Ying, Ning Li Wang

**Affiliations:** ^1^Beijing Institute of Ophthalmology, Beijing Tongren Hospital, Capital Medical University, Beijing, China; ^2^Department of Ophthalmology, University of California, San Francisco, San Francisco, CA 94143, USA; ^3^Ophthalmology Section, Surgical Service, San Francisco Veterans Affairs Medical Center, San Francisco, CA, USA; ^4^Department of Ophthalmology, Perelman School of Medicine, University of Pennsylvania, Philadelphia, PA, USA

## Abstract

**Objective:**

To estimate the prevalence rate of ocular symptoms and the positive rate of conjunctival swab samples of patients diagnosed with 2019 Novel Coronavirus Disease (COVID-19).

**Methods:**

We performed a systematic review and meta-analysis. A comprehensive literature search was done based on PubMed, Embase, MedRxiv, and the Cochrane Library. The primary outcomes are the prevalence rate of conjunctivitis/conjunctival congestion and the positive rate of conjunctival swab samples. Rates were expressed as proportions with 95% confidence intervals (CIs).

**Results:**

A total of 12 studies with 1930 participants were included for meta-analysis. The pooled prevalence rate of conjunctivitis/conjunctival congestion was 8% (95% CI: 5%-12%). 1% (95% CI: 1%-4%) of COVID-19 patients were diagnosed with conjunctivitis/conjunctival congestion as the initial symptom. The pooled positive rate of conjunctival swab samples was 3% (95% CI: 2%-5%). We also assessed other ocular symptoms reported in the 12 studies, including foreign body sensation, increased secretion, and eye itching. The pooled prevalence rates were 6% (95% CI: 3%-10%), 10% (95% CI: 8%-12%), and 9% (95% CI: 7%-10%), respectively.

**Conclusions:**

The evidence on the positive rate of conjunctival swab samples and the prevalence rates of ocular symptoms indicated that COVID-19 ocular transmission was possible but less likely.

## 1. Introduction

Humans have battled viral infections throughout history. From measles and smallpox outbreaks to the 1918 influenza pandemic, countless lives have been lost. In December 2019, a viral pneumonia of unknown etiology quickly spread worldwide. The World Health Organization declared the disease 2019 Novel Coronavirus Disease a global pandemic that is caused by severe acute respiratory syndrome coronavirus 2 (SARS-CoV2) [1]. Through 8 Sep 2020, the cumulative number of infected people stood at more than 27.25 million globally with over 891,285 deaths.

During the COVID-19 pandemic, two cases drew the attention of ophthalmologists. In one, a Chinese ophthalmologist, Dr. Wenliang Li, who raised the earliest alarm about COVID-19 in China, unfortunately died from this disease. It is not known whether contacting the eyes of patients who had contracted COVID-19 played a role in his own infection [2]. In another case, Dr. Guangfa Wang, a Chinese pneumonologist, was infected with COVID-19 in Wuhan while wearing full personal protection equipment (PPE) at all times except goggles. He complained of redness of the eyes and speculated that the virus might have attacked him via the ocular surface [3]. Through today, whether COVID-19 can transmit through the ocular surface is still controversial. Herein, we performed a comprehensive systematic review and meta-analysis to quantitatively analyze current evidence on COVID-19 ocular transmission.

## 2. Methods

### 2.1. Search Strategy and Selection Criteria

Four English electronic data sources (PubMed, Embase, MedRxiv, and the Cochrane Library) were comprehensively searched following PRISMA guidelines (from 1 December 2019 to 7 Sep 2020), and only studies in English would be screened. The following search terms and their combinations were used: “COVID-19,” “2019-nCoV-2,” “coronavirus,” “SARS-CoV-2,” “novel coronavirus,” “ocular manifestation,” “ocular symptom,” “conjunctival swab,” “Conjunctivitis,” “Conjunctival congestion.”

The eligibility of studies was independently determined by two authors, and discrepancies in study selection were resolved by team discussion.

### 2.2. Inclusion Criteria

All studies included in the meta-analysis met the following criteria: (1) the study population consisted of COVID-19 patients; (2) COVID-19 patients were diagnosed using a laboratory method; and (3) at least one of the two primary outcomes (conjunctivitis/conjunctival congestion, positive conjunctival swab samples) was assessed, and the number of events was reported.

### 2.3. Exclusion Criteria

Studies were excluded if (1) they included no data on ocular manifestations and no detection of viral RNA in conjunctival swab samples; (2) they involved animal or in vitro research; or (3) the study design was a small case report, letter, editorial, or review.

### 2.4. Data Extraction

A predefined Excel form was utilized for data collection. The following information was extracted: the first author's name, publication year, country or area where the study was conducted, sample size, mean age of subjects (in some studies, only median age or range of age was reported), and the method used to diagnose COVID-19. Most importantly, we extracted the number of events of ocular symptoms (conjunctivitis/conjunctival congestion, foreign body sensation, increased secretion, and eye itching) and the number of positive viral RNA detections in conjunctival swab samples. Any disagreement was resolved by consensus.

### 2.5. Statistical Analysis

The pooled prevalence rates of ocular symptoms, such as conjunctivitis/conjunctival congestion, were expressed using proportions with 95% confidence intervals (CIs) estimated from either a fixed-effect model or a random-effect model. Model selection was decided by the heterogeneity across included studies. The *I*^2^ statistic was used to measure the heterogeneity quantitatively. *I*^2^ describes the percentage of variability that was caused by heterogeneity rather than chance. If the *I*^2^ was below 50%, a fixed-effect model was applied, otherwise a random-effect model was used [4]. The Egger's test was used to check publication bias [5]. Sensitivity analysis was performed to explore the potential source of heterogeneity, and pooled estimations by sensitivity analysis were reported as the final results. A two-tailed *p* < 0.05 was considered statistically significant. All statistical analyses were conducted with the open source R software, version 4.0.0.

## 3. Results

### 3.1. Study Selection and Study Characterization

**Figure 1:**
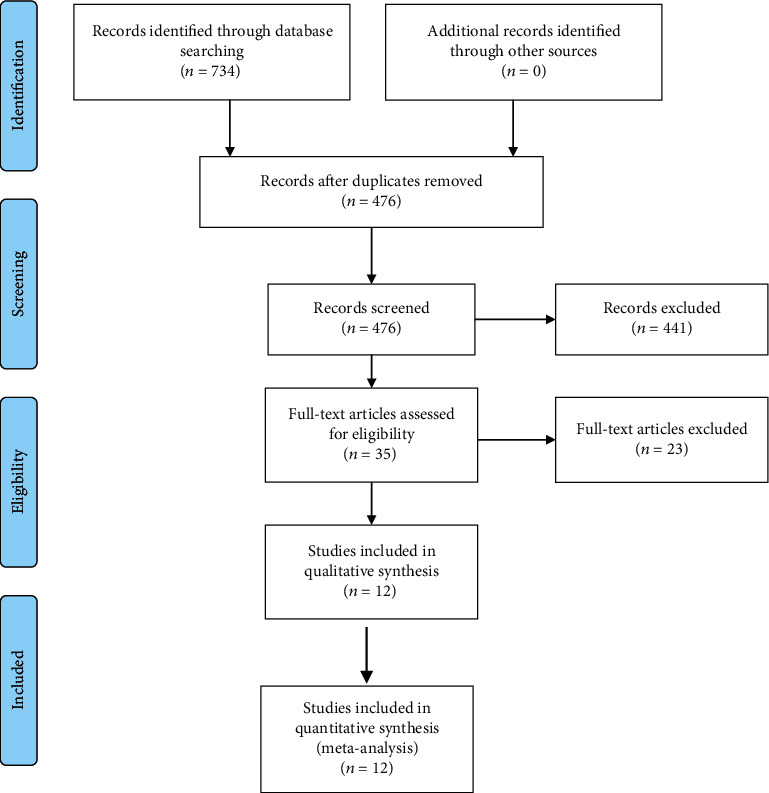
Flow chart of paper selection.

A total of 734 articles were identified after an initial database search. Of these, 258 duplications were removed and another 441 publications were further excluded by screening the title and abstract. Subsequently, 35 full-text records were evaluated for eligibility. After screening the full text, 23 articles were excluded (Figure 1). Finally, 12 studies [6–19] involving 1930 participants were included for meta-analysis.

Table 1 describes the detailed characteristics of the included studies. Eight studies were carried out in China and one in Iran. The sample size varied from 30 to 534. Most studies enrolled patients with a mean age of around 50 years old. The assessed ocular symptoms included conjunctivitis/conjunctival congestion (11 studies), foreign body sensation (six studies), increased secretion (three studies), and eye itching (five studies). All included studies used reverse transcription-polymerase chain reaction (RT-PCR) to make a diagnosis of COVID-19. Six studies reported the number of positive conjunctival swab samples.

### 3.2. Systematic Review and Meta-Analysis of Ocular Symptoms

**Figure 2:**
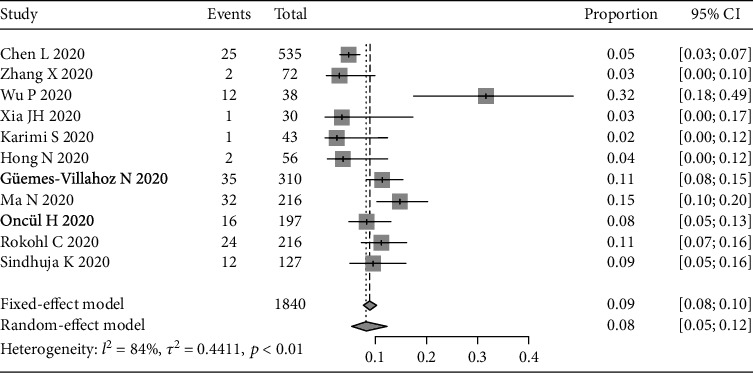
Forest plot of conjunctivitis/conjunctival congestion.

The pooled prevalence rate of conjunctivitis/conjunctival congestion estimated by the random-effect model was 8% (95% CI: 5%-12%, Figure 2) because the heterogeneity was relatively large (*I*^2^ = 84%, *p* < 0.01). We further performed a sensitivity analysis. After the study of Wu et al. in 2020 [15] was omitted, the *I*^2^ dropped sharply, indicating that the study of Wu et al. in 2020 was the source of heterogeneity. The reason was that Wu et al. took all the following symptoms into account when defining conjunctivitis: conjunctival hyperemia, chemosis, epiphora, and increased secretion. This resulted in a much higher prevalence rate of conjunctivitis/conjunctival congestion than those of the other studies. The pooled prevalence rate of conjunctivitis/conjunctival congestion was 8.3% (95% CI: 7.1%-9.7%) by sensitivity analysis.

**Figure 3:**
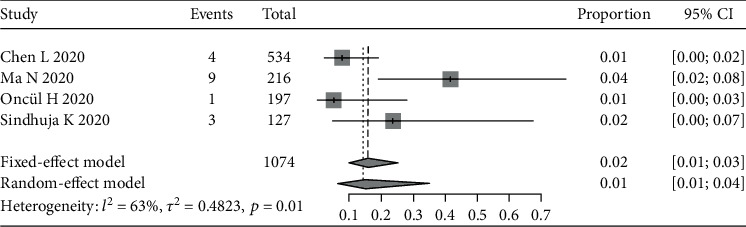
Forest plot of conjunctivitis/conjunctival congestion as initial symptom.

Notably, four studies [6, 10–12, 14] reported 17 COVID-19 patients whose initial symptom was conjunctivitis/conjunctival congestion (Figure 3). The pooled prevalence rate was 1% (95% CI: 1%-4%). The corresponding *I*^2^ was 63% across the four studies.

**Figure 4:**
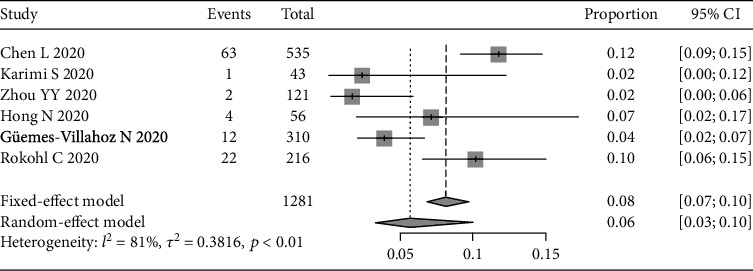
Forest plot of foreign body sensation.

**Figure 5:**
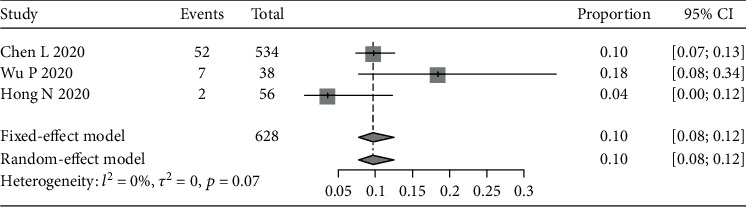
Forest plot of increased secretion.

**Figure 6:**
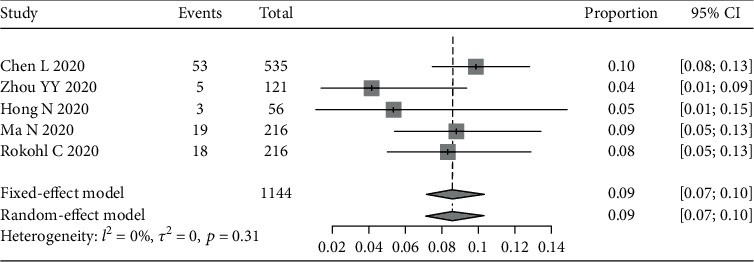
Forest plot of eye itching.

We also assessed other ocular symptoms reported in the 12 studies, including foreign body sensation (see Figure 4), increased secretion (see Figure 5), and eye itching (see Figure 6). The pooled prevalence rates were 6% (95% CI: 3%-10%), 10% (95% CI: 8%-12%), and 9% (95% CI: 7%-10%), respectively.

A heterogeneity test of the pooled prevalence rate of foreign body sensation showed a relatively large heterogeneity (*I*^2^ = 81%, *p* < 0.01), thus a sensitivity analysis was applied. The pooled prevalence rate of foreign body sensation was 5.5% (95% CI: 4.1%-7.4%).

### 3.3. Detection of Viral RNA in Conjunctival Swab Samples

**Figure 7:**
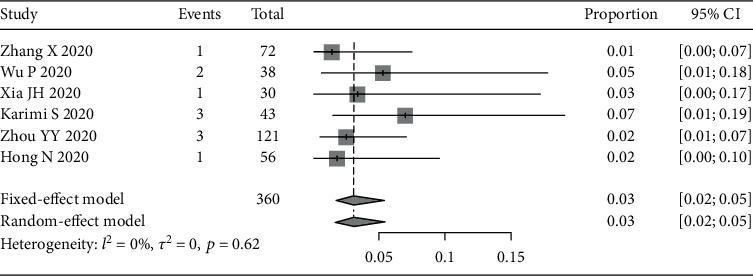
Forest plot of positive rate of conjunctival swab samples.

Six studies reported positive conjunctival swab samples. The pooled positive rate of conjunctival swab samples was estimated using a fixed-effect model (Figure 7) since there was no heterogeneity across the six studies (*I*^2^ = 0%, *p* = 0.62). The pooled positive rate of conjunctival swab samples was 3% (95% CI: 2%-5%).

Results of Egger's test (see Table 2) showed no publication bias for the following models: the pooled prevalence rate of conjunctivitis/conjunctival congestion (*t* = −0.975, *p* = 0.355), the pooled prevalence rate of conjunctivitis/conjunctival congestion as initial symptom (*t* = −1.515, *p* = 0.269), the pooled positive events of conjunctival swab samples (*t* = −1.242, *p* = 0.282), the pooled prevalence rate of foreign body sensation (*t* = −2.652, *p* = 0.057), and increased secretion (*t* = −0.063, *p* = 0.960). Egger's test indicated that there might be a publication bias in the pooled prevalence rate of eye itching (*t* = −4.263, *p* = 0.024).

## 4. Discussion

This systematic review and meta-analysis is aimed at quantitatively evaluating the current evidence for the possibility of COVID-19 ocular transmission. We identified and analyzed 12 studies with 1930 COVID-19 participants. The common ocular symptoms involved conjunctivitis/conjunctival congestion, increased secretions, eye itching, and foreign body sensation. Interestingly, 1% of COVID-19 patients were diagnosed with conjunctivitis/conjunctival congestion as the initial symptom. In addition, our meta-analysis revealed that the pooled positive rate of conjunctival swab samples was 3%. These results suggested that ocular transmission of the COVID-19 virus was possible but unlikely.

Laboratory studies had been conducted to explore the mechanisms of COVID-19 ocular transmission. The SARS-CoV-2 infection has a high degree of similarity to SARS-CoV, which infects the host receptor of angiotensin-converting enzyme 2 (ACE2), resulting in cross-species and human-to-human transmissions [20]. During the SARS pandemic, a study confirmed that ACE2 was a functional receptor for SARS-CoV [21]. A recent study revealed that ACE2 is expressed in human cornea tissues and that a high and consistent expression of ACE2 in the cornea poses a high potential for infection by SARS-CoV-2 [10, 11]. ACE2 expression has also been reported in human aqueous humor [22–24]. A recent genomics study demonstrated that the ACE2 gene was expressed in corneal epithelial cells, which suggests that the eyes could be vulnerable to COVID-19 infection [25].

In addition, from the perspective of human anatomical features, the nasolacrimal system forms a bridge between the ocular surface and the respiratory tract. Therefore, conjunctival secretions containing the virus can drain into the nasopharyngeal space and infect the human body. Consistent with this perspective, a recent study revealed that rhesus macaques can be infected with COVID-19 through ocular conjunctival inoculation [26].

In support of the laboratory data, much clinical evidence indicates the possibility of ocular surface transmission. Studies have reported ocular manifestation as the first sign of COVID-19 in patients [27–29]. In one COVID-19 case [29], conjunctivitis was even reported to be the only presenting sign and symptom of COVID-19. Recently, Xia et al. isolated SARS-CoV-2 in tears [16]. Similarly, our meta-analysis found that up to 10% of COVID-19 patients had ocular symptoms and 3% of conjunctival swab samples were positive in COVID-19 patients, supporting the possibility of transmission through the ocular surface.

However, the prevalence rates of ocular symptoms and the positive rate of conjunctival swab samples were relatively low based on our meta-analysis. Meanwhile, studies reported that 94% to 98% of COVID-19 patients showed fever [18, 19], 79% showed cough [18, 19], and 44% showed myalgia or fatigue [30]; these manifestations of COVID-19 were more common than ocular manifestations. The current evidence indicates that the risk of contracting COVID-19 via ocular tissue is relatively low, although this could be due to limitations of current detection methods.

The strength of our study is that it is the first meta-analysis to summarize the rapidly emerging yet controversial publications reporting the prevalence rates of ocular symptoms and the positive rate of conjunctival swab samples in COVID-19 patients. However, there are several limitations of our meta-analysis. Firstly, in many studies, it was difficult to determine whether patients' ocular symptoms showed up before or after they were diagnosed with COVID-19, which might cause bias in the estimation of the prevalence rates. Secondly, ocular symptoms were reported in some but not all studies, which could lead to relatively low pooled prevalence rates.

In summary, this meta-analysis showed that the pooled prevalence rate of conjunctivitis/conjunctival congestion was 8% in patients with COVID-19. About one percent of COVID-19 patients were diagnosed with conjunctivitis/conjunctival congestion as the initial symptom. The pooled positive rate of conjunctival swab samples was 3%. Ocular transmission of COVID-19 may be possible but seems unlikely; however, it still might be worthy for ophthalmologists to wear protective eye goggles to minimize the risk of ocular transmission.

## Figures and Tables

**Table 1:** Characteristics of included studies for meta-analysis.

First author	Publication year	Country/area	Sample size	Mean age (years)	Ocular symptoms	Conjunctivitis/conjunctival congestion	Conjunctivitis as initial symptom	Foreign body sensation	Increased secretions	Eye itching	Positive conjunctival swab samples	Laboratory method
Chen L	2020	China	534	Range: 16-68	27	27	4	63	52	53	**—**	RT-PCR
Zhang X	2020	China	72	58.7 ± 14.8	2	2	—	—	—	—	**1**	RT-PCR
Wu P	2020	China	38	65.8 ± 16.6	12	12	—	—	7	—	**2**	RT-PCR
Xia JH	2020	China	30	54.5 ± 14.2	1	1	—	—	—	—	**1**	RT-PCR
Karimi S	2020	Iran	43	56 ± 13	2	1	—	1	—	—	**3**	RT-PCR
Zhou YY	2020	China	121	Median: 48 (range: 22-89)	8	—	—	2	—	5	**3**	RT-PCR
Hong N	2020	China	56	48 ± 12.1	15	2	—	4	2	3	**1**	RT-PCR
Guemes-Villahoz N	2020	Spain	310	Median: 72 (IQR: 59-82)	35	35	—	12	—	—	—	RT-PCR
Ma N	2020	China	216	Median: 7.25 (range: 2.6-11.6)	59	32	9	—	—	19	—	RT-PCR
Öncül H	2020	Turkey	197	Median: 58.5 (range: 20-91)	16	8	1	—	—	—	—	RT-PCR
Rokohl C	2020	Germany	216	37.9 ± 13.7	—	24	—	22	—	56	—	RT-PCR
Sindhuja K	2020	India	127	Median: 38.8	12	11	3	—	—	—	—	RT-PCR

RT-PCR: reverse transcription-polymerase chain reaction; IQR: interquartile range.

**Table 2:** Results of publication bias by Egger's test.

Tested model	*t*	*p*
Figure 2	-0.975	0.355
Figure 3	-1.515	0.269
Figure 7	-1.242	0.282
Figure 4	-2.652	0.057
Figure 5	-0.063	0.960
Figure 6	-4.263	0.024
